# Supplementary material for: Nkx2-5 defines distinct scaffold and recruitment phases during formation of the murine cardiac Purkinje fiber network
Source: Nat Commun. 2020 Oct 20;11:5300. doi: 10.1038/s41467-020-19150-9 (PMC7575572; doi:10.1038/s41467-020-19150-9)
Supplement: Supplementary file 1 — Supplementary Information [file 41467_2020_19150_MOESM1_ESM.pptx]

## Slide 1
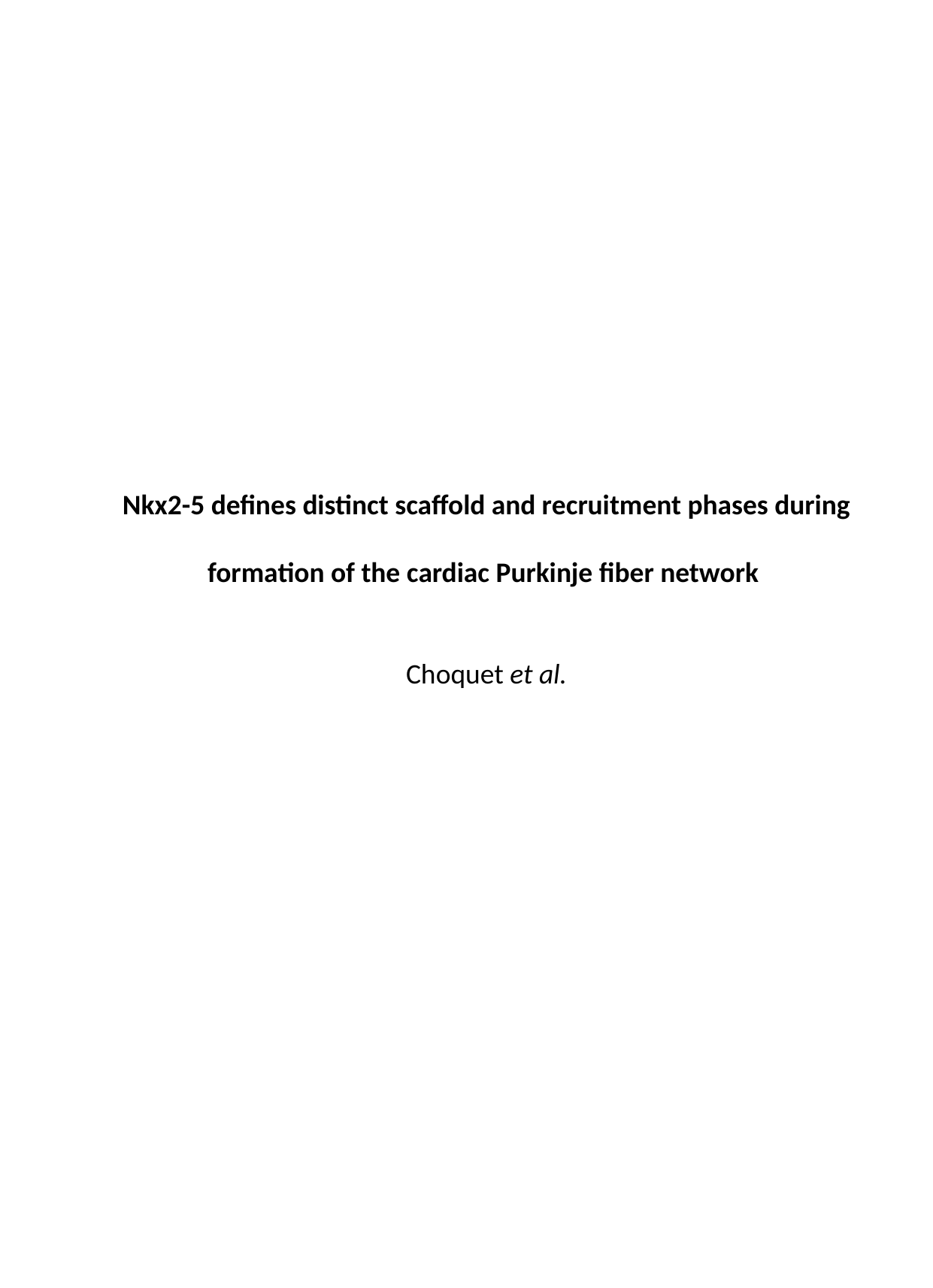

Nkx2-5 defines distinct scaffold and recruitment phases during formation of the cardiac Purkinje fiber network
Choquet et al.

## Slide 2
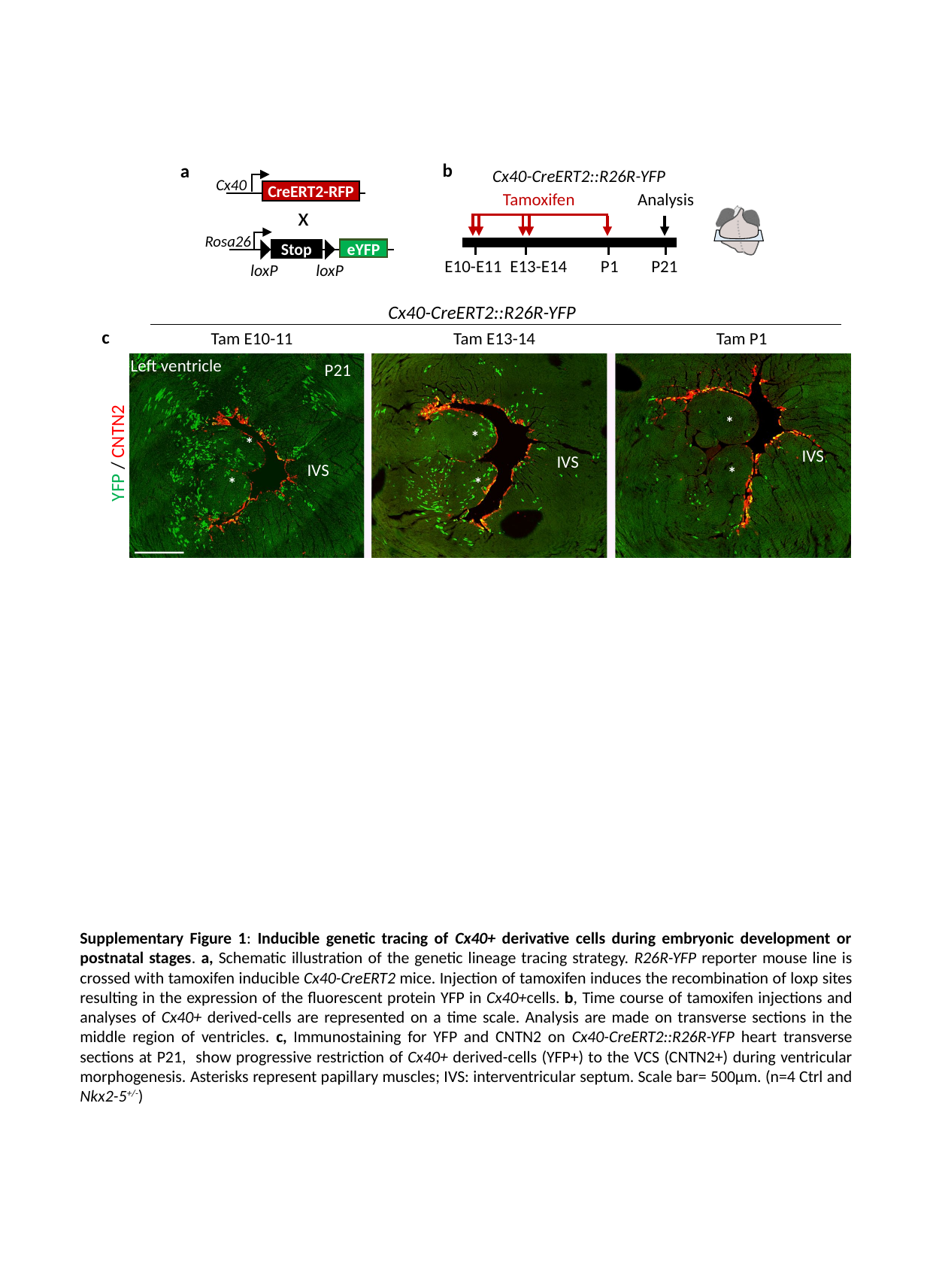

b
Cx40-CreERT2::R26R-YFP
Analysis
Tamoxifen
E10-E11
P21
P1
E13-E14
a
Cx40
CreERT2-RFP
x
Rosa26
eYFP
Stop
loxP
loxP
Cx40-CreERT2::R26R-YFP
Tam E13-14
Tam P1
Tam E10-11
*
*
*
*
*
*
YFP / CNTN2
P21
Left ventricle
IVS
IVS
IVS
c
Supplementary Figure 1: Inducible genetic tracing of Cx40+ derivative cells during embryonic development or postnatal stages. a, Schematic illustration of the genetic lineage tracing strategy. R26R-YFP reporter mouse line is crossed with tamoxifen inducible Cx40-CreERT2 mice. Injection of tamoxifen induces the recombination of loxp sites resulting in the expression of the fluorescent protein YFP in Cx40+cells. b, Time course of tamoxifen injections and analyses of Cx40+ derived-cells are represented on a time scale. Analysis are made on transverse sections in the middle region of ventricles. c, Immunostaining for YFP and CNTN2 on Cx40-CreERT2::R26R-YFP heart transverse sections at P21, show progressive restriction of Cx40+ derived-cells (YFP+) to the VCS (CNTN2+) during ventricular morphogenesis. Asterisks represent papillary muscles; IVS: interventricular septum. Scale bar= 500µm. (n=4 Ctrl and Nkx2-5+/-)

## Slide 3
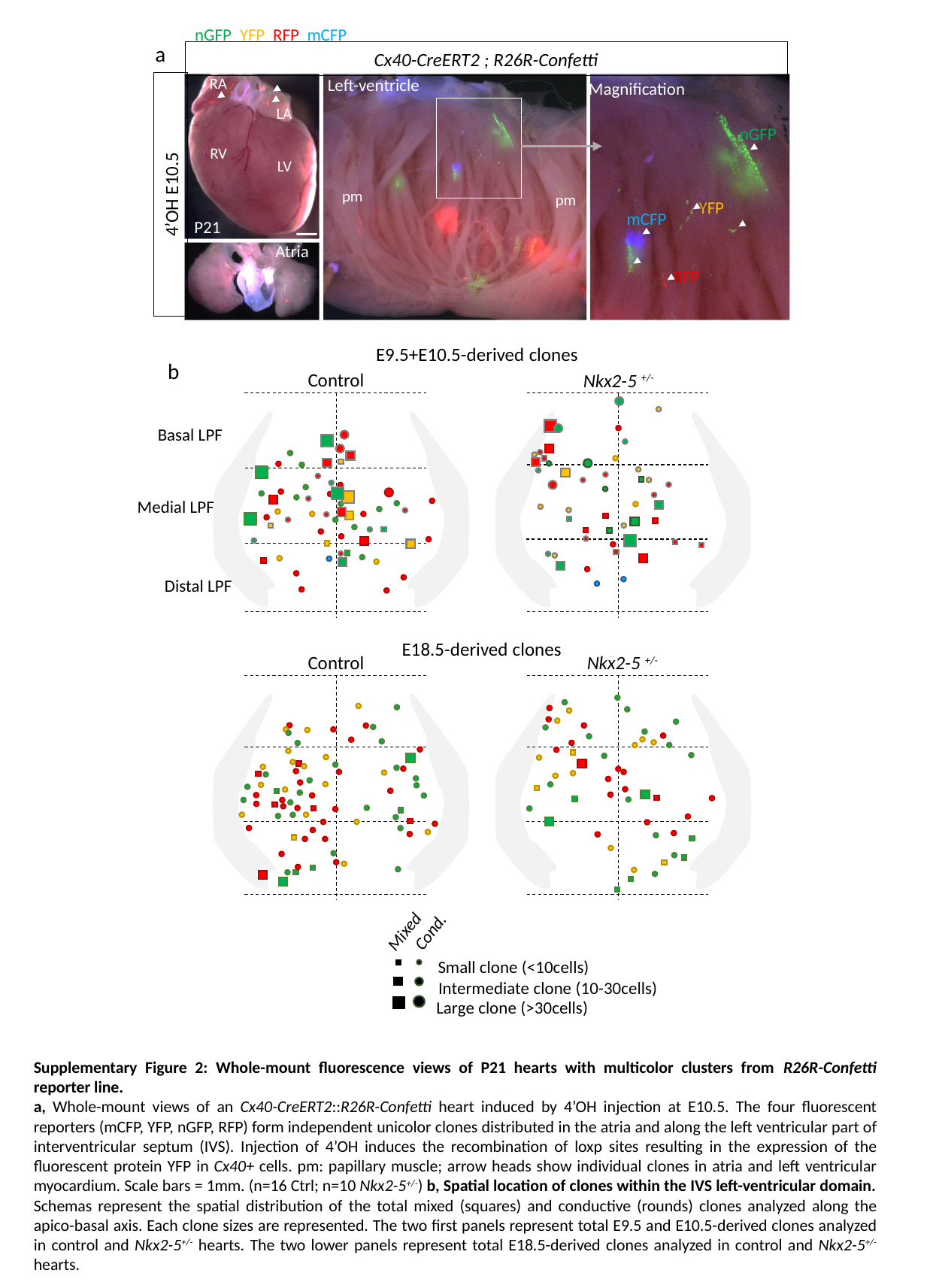

nGFP YFP RFP mCFP
a
RA
LA
RV
LV
Left-ventricle
Magnification
nGFP
pm
pm
YFP
mCFP
Atria
RFP
P21
Cx40-CreERT2 ; R26R-Confetti
4’OH E10.5
E9.5+E10.5-derived clones
Control
Basal LPF
Medial LPF
Distal LPF
Nkx2-5 +/-
E18.5-derived clones
Control
Nkx2-5 +/-
b
P21
P21
RA
Cond.
Small clone (<10cells)
Intermediate clone (10-30cells)
Large clone (>30cells)
Mixed
LA
Supplementary Figure 2: Whole-mount fluorescence views of P21 hearts with multicolor clusters from R26R-Confetti reporter line.
a, Whole-mount views of an Cx40-CreERT2::R26R-Confetti heart induced by 4’OH injection at E10.5. The four fluorescent reporters (mCFP, YFP, nGFP, RFP) form independent unicolor clones distributed in the atria and along the left ventricular part of interventricular septum (IVS). Injection of 4’OH induces the recombination of loxp sites resulting in the expression of the fluorescent protein YFP in Cx40+ cells. pm: papillary muscle; arrow heads show individual clones in atria and left ventricular myocardium. Scale bars = 1mm. (n=16 Ctrl; n=10 Nkx2-5+/-) b, Spatial location of clones within the IVS left-ventricular domain. Schemas represent the spatial distribution of the total mixed (squares) and conductive (rounds) clones analyzed along the apico-basal axis. Each clone sizes are represented. The two first panels represent total E9.5 and E10.5-derived clones analyzed in control and Nkx2-5+/- hearts. The two lower panels represent total E18.5-derived clones analyzed in control and Nkx2-5+/- hearts.

## Slide 4
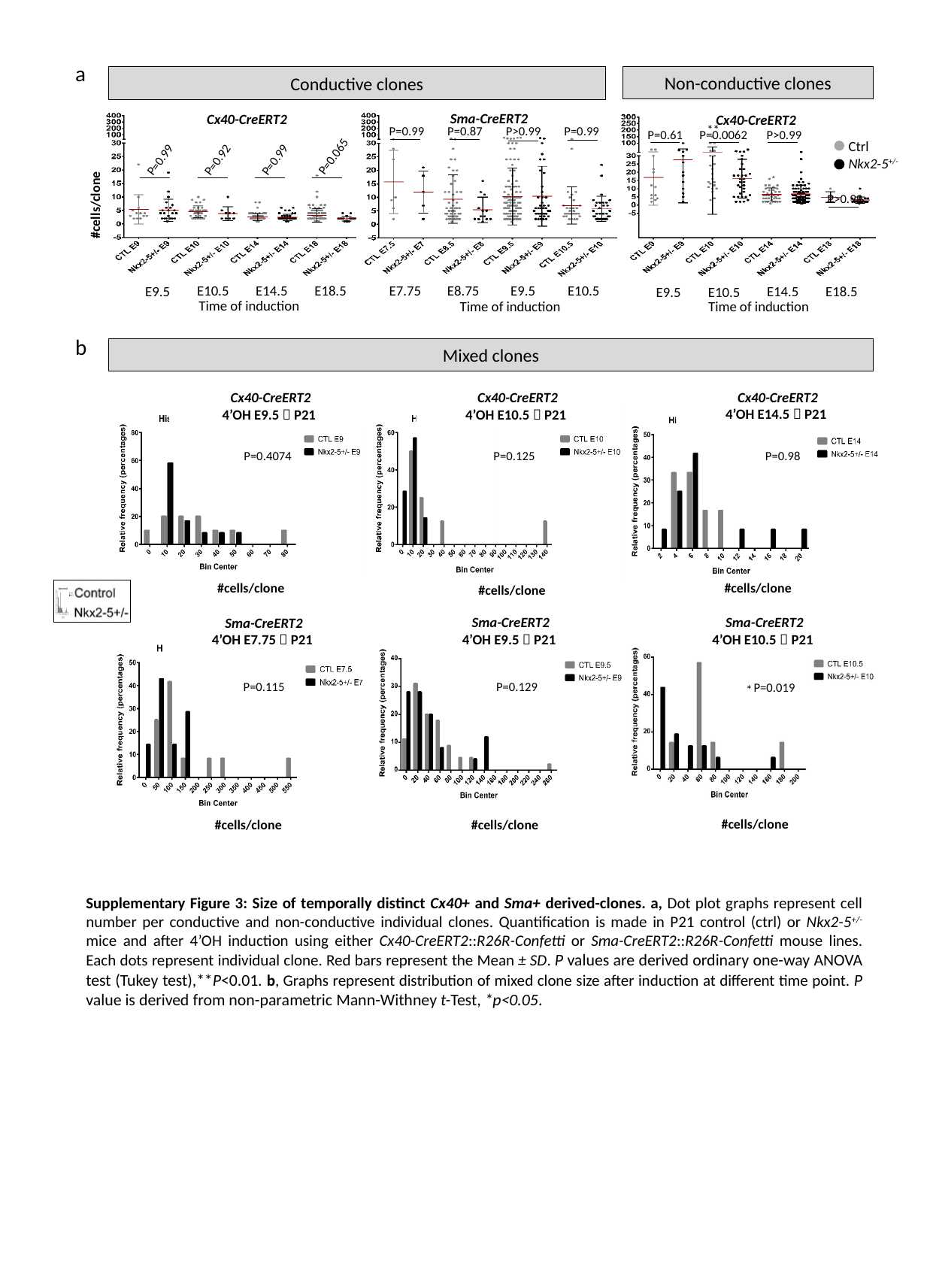

a
Non-conductive clones
Conductive clones
Sma-CreERT2
Cx40-CreERT2
Cx40-CreERT2
Ctrl
Nkx2-5+/-
E14.5
E18.5
E10.5
E9.5
E7.75
E10.5
E8.75
E9.5
E14.5
E18.5
E10.5
E9.5
Time of induction
Time of induction
Time of induction
**
P=0.99
P=0.87
P>0.99
P=0.99
P=0.0062
P=0.61
P>0.99
P=0.065
P=0.99
P=0.92
P=0.99
#cells/clone
P>0.99
b
Mixed clones
Cx40-CreERT2
4’OH E14.5  P21
Cx40-CreERT2
4’OH E9.5  P21
Cx40-CreERT2
4’OH E10.5  P21
#cells/clone
#cells/clone
#cells/clone
Sma-CreERT2
4’OH E9.5  P21
Sma-CreERT2
4’OH E10.5  P21
Sma-CreERT2
4’OH E7.75  P21
4’OH E10.5  P21
#cells/clone
#cells/clone
#cells/clone
P=0.4074
P=0.125
P=0.98
P=0.115
P=0.129
P=0.019
*
Supplementary Figure 3: Size of temporally distinct Cx40+ and Sma+ derived-clones. a, Dot plot graphs represent cell number per conductive and non-conductive individual clones. Quantification is made in P21 control (ctrl) or Nkx2-5+/- mice and after 4’OH induction using either Cx40-CreERT2::R26R-Confetti or Sma-CreERT2::R26R-Confetti mouse lines. Each dots represent individual clone. Red bars represent the Mean ± SD. P values are derived ordinary one-way ANOVA test (Tukey test),**P<0.01. b, Graphs represent distribution of mixed clone size after induction at different time point. P value is derived from non-parametric Mann-Withney t-Test, *p<0.05.

## Slide 5
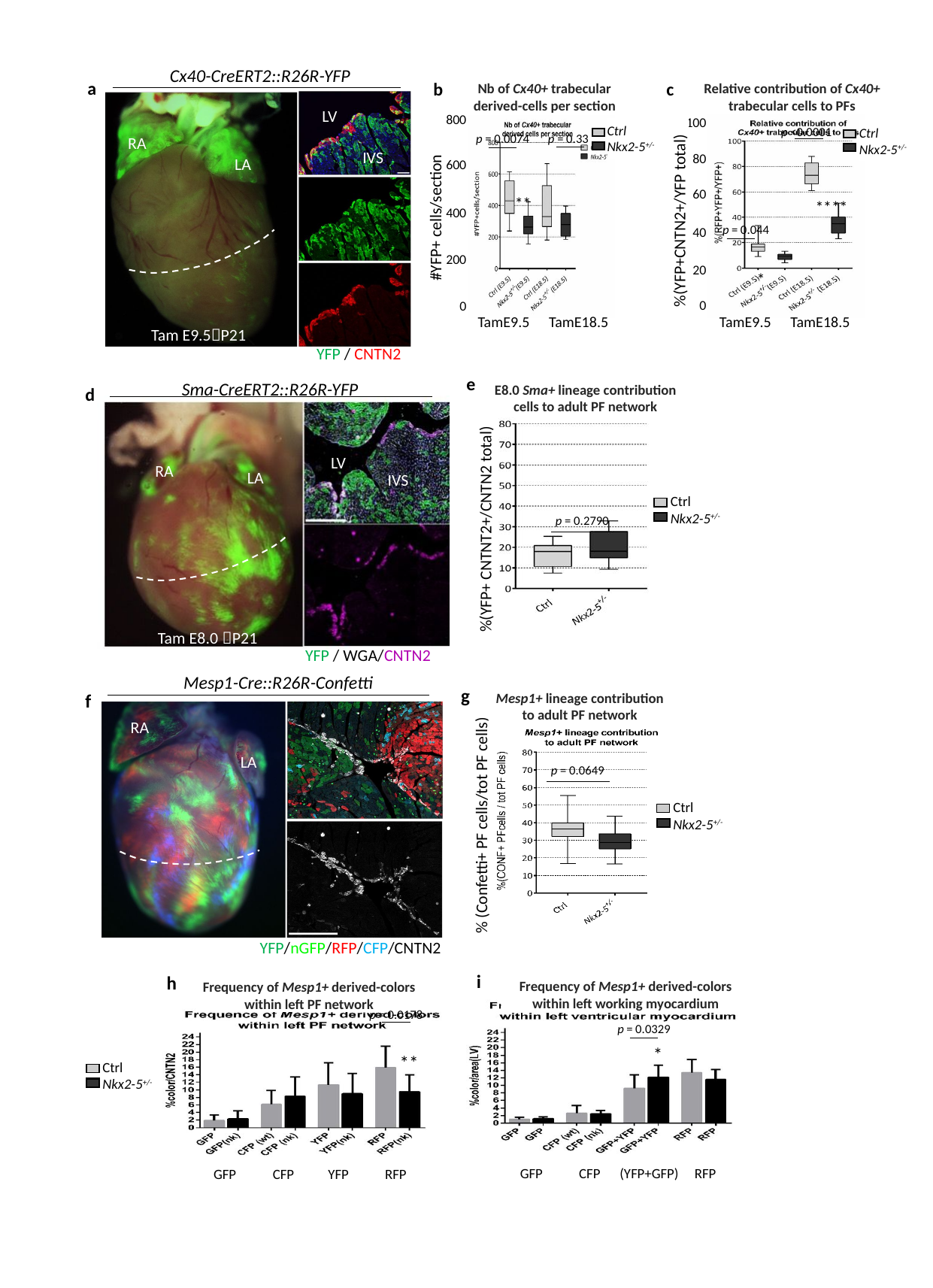

Cx40-CreERT2::R26R-YFP
a
LV
IVS
YFP / CNTN2
Tam E9.5P21 P21
RA
LA
b
**
Nb of Cx40+ trabecular derived-cells per section
800
600
400
200
0
#YFP+ cells/section
TamE9.5
TamE18.5
Ctrl
Nkx2-5+/-
c
Relative contribution of Cx40+ trabecular cells to PFs
TamE9.5
TamE18.5
100
80
60
40
20
0
Ctrl
Nkx2-5+/-
****
*
%(YFP+CNTN2+/YFP total)
p <0.0001
p = 0.33
p = 0.0074
p = 0.044
e
Sma-CreERT2::R26R-YFP
d
LV
RA
LA
IVS
Tam E8.0 P21
YFP / WGA/CNTN2
E8.0 Sma+ lineage contribution cells to adult PF network
%(YFP+ CNTNT2+/CNTN2 total)
Ctrl
Nkx2-5+/-
p = 0.2790
Mesp1-Cre::R26R-Confetti
g
f
Mesp1+ lineage contribution to adult PF network
Ctrl
Nkx2-5+/-
% (Confetti+ PF cells/tot PF cells)
RA
LA
YFP/nGFP/RFP/CFP/CNTN2
p = 0.0649
i
Frequency of Mesp1+ derived-colors within left working myocardium
GFP
CFP
(YFP+GFP)
RFP
h
Frequency of Mesp1+ derived-colors within left PF network
GFP
CFP
YFP
RFP
*
**
Ctrl
Nkx2-5+/-
p = 0.0178
p = 0.0329

## Slide 6
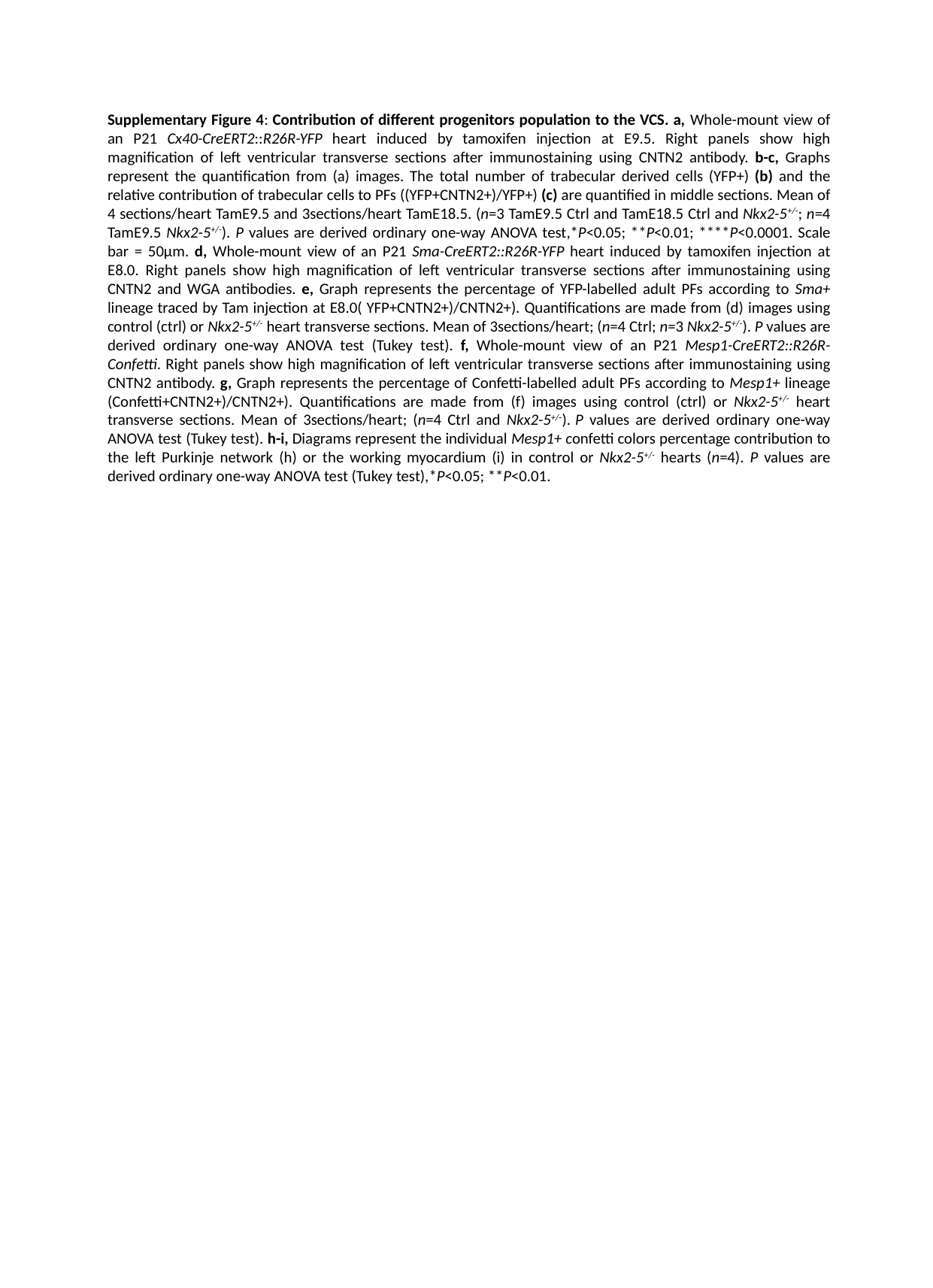

Supplementary Figure 4: Contribution of different progenitors population to the VCS. a, Whole-mount view of an P21 Cx40-CreERT2::R26R-YFP heart induced by tamoxifen injection at E9.5. Right panels show high magnification of left ventricular transverse sections after immunostaining using CNTN2 antibody. b-c, Graphs represent the quantification from (a) images. The total number of trabecular derived cells (YFP+) (b) and the relative contribution of trabecular cells to PFs ((YFP+CNTN2+)/YFP+) (c) are quantified in middle sections. Mean of 4 sections/heart TamE9.5 and 3sections/heart TamE18.5. (n=3 TamE9.5 Ctrl and TamE18.5 Ctrl and Nkx2-5+/-; n=4 TamE9.5 Nkx2-5+/-). P values are derived ordinary one-way ANOVA test,*P<0.05; **P<0.01; ****P<0.0001. Scale bar = 50µm. d, Whole-mount view of an P21 Sma-CreERT2::R26R-YFP heart induced by tamoxifen injection at E8.0. Right panels show high magnification of left ventricular transverse sections after immunostaining using CNTN2 and WGA antibodies. e, Graph represents the percentage of YFP-labelled adult PFs according to Sma+ lineage traced by Tam injection at E8.0( YFP+CNTN2+)/CNTN2+). Quantifications are made from (d) images using control (ctrl) or Nkx2-5+/- heart transverse sections. Mean of 3sections/heart; (n=4 Ctrl; n=3 Nkx2-5+/-). P values are derived ordinary one-way ANOVA test (Tukey test). f, Whole-mount view of an P21 Mesp1-CreERT2::R26R-Confetti. Right panels show high magnification of left ventricular transverse sections after immunostaining using CNTN2 antibody. g, Graph represents the percentage of Confetti-labelled adult PFs according to Mesp1+ lineage (Confetti+CNTN2+)/CNTN2+). Quantifications are made from (f) images using control (ctrl) or Nkx2-5+/- heart transverse sections. Mean of 3sections/heart; (n=4 Ctrl and Nkx2-5+/-). P values are derived ordinary one-way ANOVA test (Tukey test). h-i, Diagrams represent the individual Mesp1+ confetti colors percentage contribution to the left Purkinje network (h) or the working myocardium (i) in control or Nkx2-5+/- hearts (n=4). P values are derived ordinary one-way ANOVA test (Tukey test),*P<0.05; **P<0.01.

## Slide 7
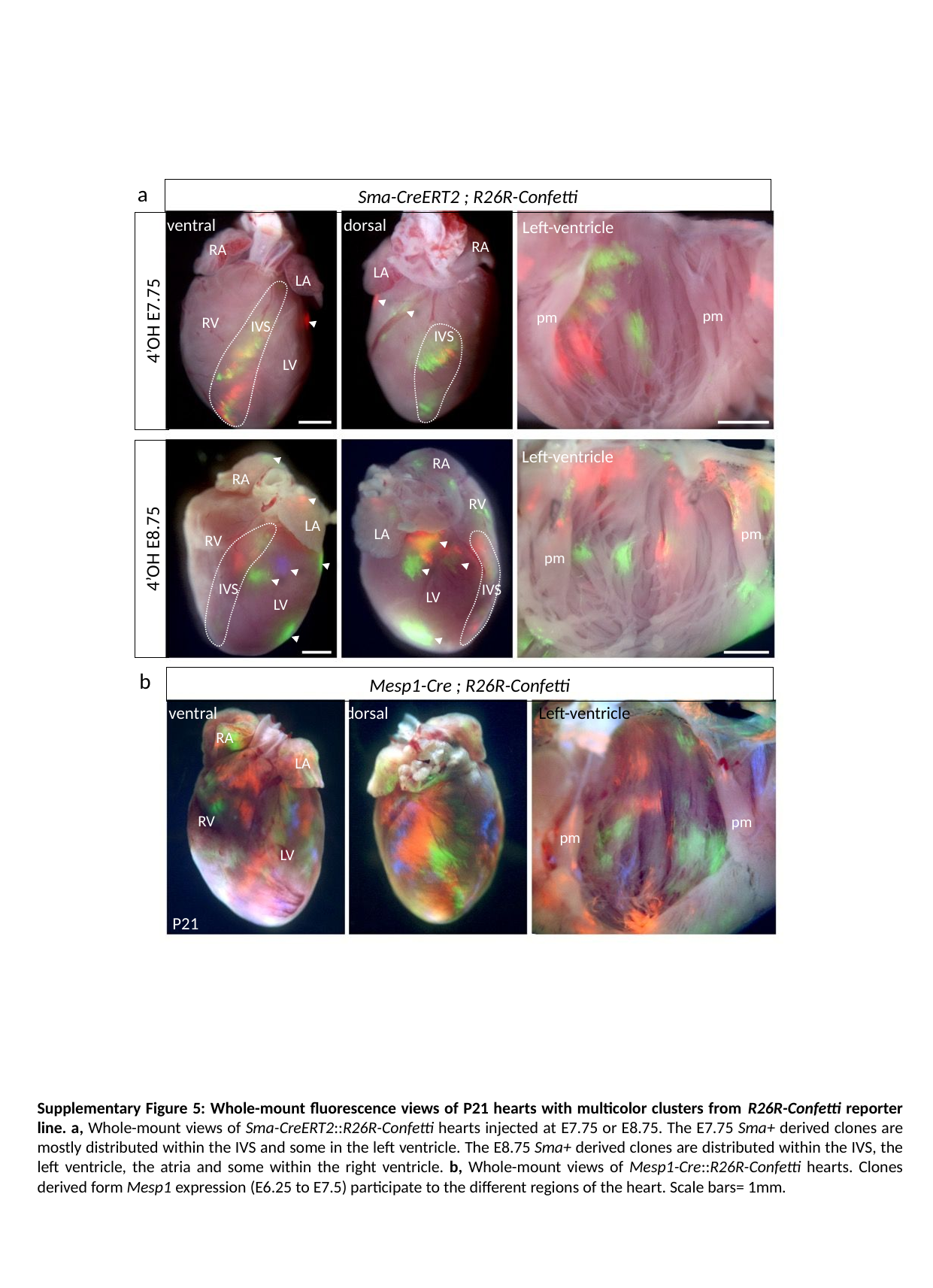

a
Sma-CreERT2 ; R26R-Confetti
ventral
dorsal
Left-ventricle
RA
RA
LA
LA
pm
pm
RV
IVS
4’OH E7.75
IVS
LV
Left-ventricle
RA
RA
RV
LA
LA
pm
RV
4’OH E8.75
pm
IVS
IVS
LV
LV
b
Mesp1-Cre ; R26R-Confetti
ventral
Left-ventricle
dorsal
RA
LA
RV
pm
pm
LV
P21
Supplementary Figure 5: Whole-mount fluorescence views of P21 hearts with multicolor clusters from R26R-Confetti reporter line. a, Whole-mount views of Sma-CreERT2::R26R-Confetti hearts injected at E7.75 or E8.75. The E7.75 Sma+ derived clones are mostly distributed within the IVS and some in the left ventricle. The E8.75 Sma+ derived clones are distributed within the IVS, the left ventricle, the atria and some within the right ventricle. b, Whole-mount views of Mesp1-Cre::R26R-Confetti hearts. Clones derived form Mesp1 expression (E6.25 to E7.5) participate to the different regions of the heart. Scale bars= 1mm.

## Slide 8
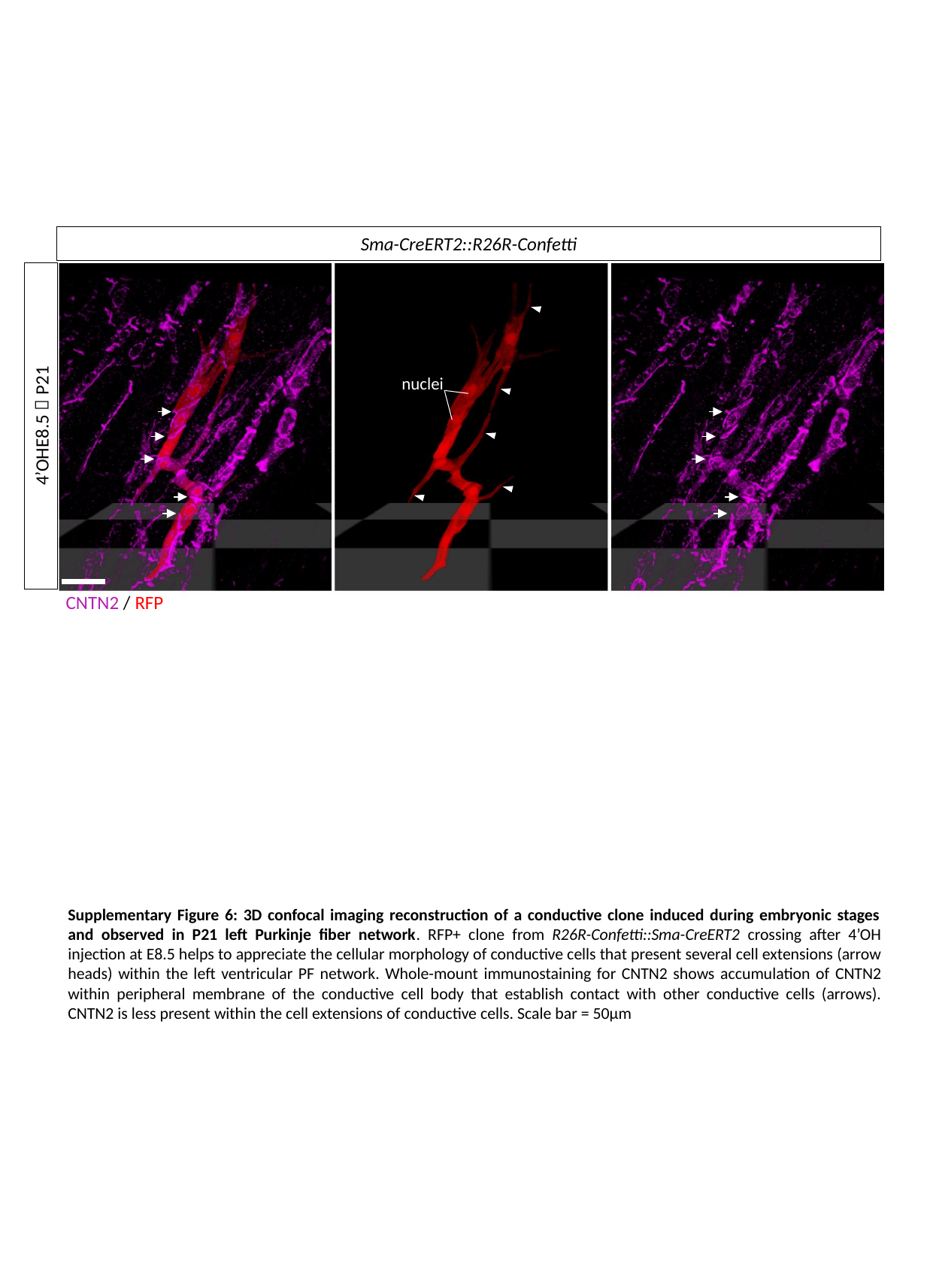

Sma-CreERT2::R26R-Confetti
nuclei
4’OHE8.5  P21
CNTN2 / RFP
Supplementary Figure 6: 3D confocal imaging reconstruction of a conductive clone induced during embryonic stages and observed in P21 left Purkinje fiber network. RFP+ clone from R26R-Confetti::Sma-CreERT2 crossing after 4’OH injection at E8.5 helps to appreciate the cellular morphology of conductive cells that present several cell extensions (arrow heads) within the left ventricular PF network. Whole-mount immunostaining for CNTN2 shows accumulation of CNTN2 within peripheral membrane of the conductive cell body that establish contact with other conductive cells (arrows). CNTN2 is less present within the cell extensions of conductive cells. Scale bar = 50µm

## Slide 9
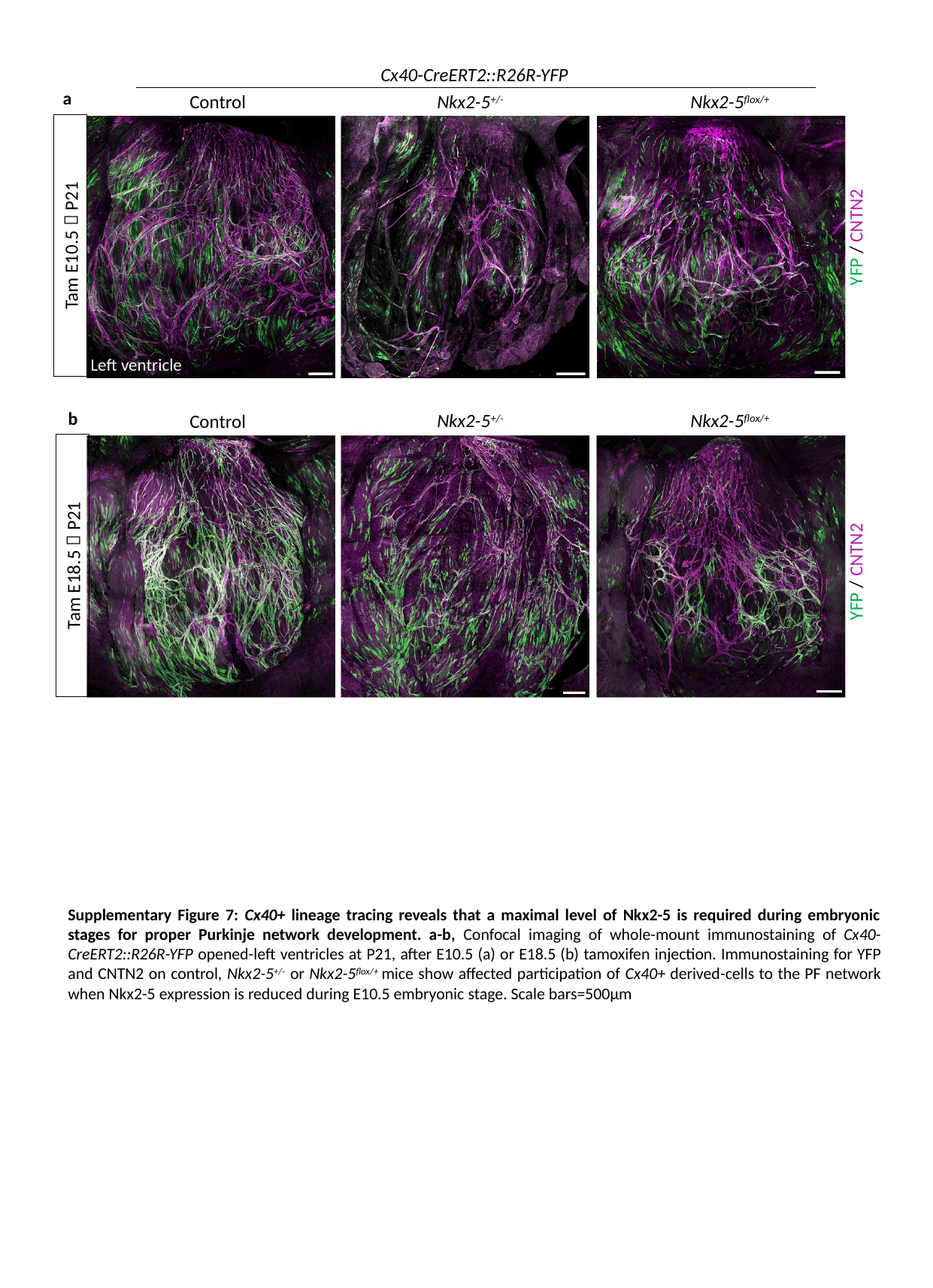

Cx40-CreERT2::R26R-YFP
a
Nkx2-5+/-
Nkx2-5flox/+
Control
YFP / CNTN2
Tam E10.5  P21
Left ventricle
b
Nkx2-5+/-
Nkx2-5flox/+
Control
Tam E18.5  P21
YFP / CNTN2
Supplementary Figure 7: Cx40+ lineage tracing reveals that a maximal level of Nkx2-5 is required during embryonic stages for proper Purkinje network development. a-b, Confocal imaging of whole-mount immunostaining of Cx40-CreERT2::R26R-YFP opened-left ventricles at P21, after E10.5 (a) or E18.5 (b) tamoxifen injection. Immunostaining for YFP and CNTN2 on control, Nkx2-5+/- or Nkx2-5flox/+ mice show affected participation of Cx40+ derived-cells to the PF network when Nkx2-5 expression is reduced during E10.5 embryonic stage. Scale bars=500µm
