# Supplementary material for: Nkx2-5 defines distinct scaffold and recruitment phases during formation of the murine cardiac Purkinje fiber network
Source: Nat Commun. 2020 Oct 20;11:5300. doi: 10.1038/s41467-020-19150-9 (PMC7575572; doi:10.1038/s41467-020-19150-9)
Supplement: Supplementary file 3 — Reporting Summary [file 41467_2020_19150_MOESM3_ESM.pdf]

nature research

Corresponding author(s): MIQUEROL Lucile

Last updated by author(s): 2020/07/31

Reporting Summary

Nature Research wishes to improve the reproducibility of the work that we publish. This form provides structure for consistency and transparency in reporting. For further information on Nature Research policies, see our Editorial Policies and the Editorial Policy Checklist.

Statistics

For all statistical analyses, confirm that the following items are present in the figure legend, table legend, main text, or Methods section.

n/a	Confirmed	
☐	☒	The exact sample size (n) for each experimental group/condition, given as a discrete number and unit of measurement
☐	☒	A statement on whether measurements were taken from distinct samples or whether the same sample was measured repeatedly
☐	☒	The statistical test(s) used AND whether they are one- or two-sided<br>*Only common tests should be described solely by name; describe more complex techniques in the Methods section.*
☒	☐	A description of all covariates tested
☐	☒	A description of any assumptions or corrections, such as tests of normality and adjustment for multiple comparisons
☐	☒	A full description of the statistical parameters including central tendency (e.g. means) or other basic estimates (e.g. regression coefficient) AND variation (e.g. standard deviation) or associated estimates of uncertainty (e.g. confidence intervals)
☐	☒	For null hypothesis testing, the test statistic (e.g. F, t, r) with confidence intervals, effect sizes, degrees of freedom and P value noted<br>*Give P values as exact values whenever suitable.*
☒	☐	For Bayesian analysis, information on the choice of priors and Markov chain Monte Carlo settings
☒	☐	For hierarchical and complex designs, identification of the appropriate level for tests and full reporting of outcomes
☒	☐	Estimates of effect sizes (e.g. Cohen's d, Pearson's r), indicating how they were calculated

Our web collection on statistics for biologists contains articles on many of the points above.

Software and code

Policy information about availability of computer code

Data collection	Wholemount images were acquired on Zeiss Lumar V12 or confocal LSM 780 microscopes and immunofluorescence sections images were acquired on Zeiss apotome Z1 using Zeiss software Zen pro 2012 blue version 1.1.2.0.
Data analysis	For processing imaging data: Adobe Photoshop 13.0.1 / Image J 1.48v /3D reconstruction and clones quantification were carried out using Volocity 5.3.1.<br>Statistics and graphs were genrated using Prism-Graphpad 6.01.

For manuscripts utilizing custom algorithms or software that are central to the research but not yet described in published literature, software must be made available to editors and reviewers. We strongly encourage code deposition in a community repository (e.g. GitHub). See the Nature Research guidelines for submitting code & software for further information.

Data

Policy information about availability of data

All manuscripts must include a data availability statement. This statement should provide the following information, where applicable:

- Accession codes, unique identifiers, or web links for publicly available datasets
- A list of figures that have associated raw data
- A description of any restrictions on data availability

All data supporting the findings of this study are available from the corresponding author on reasonable request.

Field-specific reporting

Please select the one below that is the best fit for your research. If you are not sure, read the appropriate sections before making your selection.

☒ Life sciences ☐ Behavioural & social sciences ☐ Ecological, evolutionary & environmental sciences

For a reference copy of the document with all sections, see nature.com/documents/nr-reporting-summary-flat.pdf

Life sciences study design

All studies must disclose on these points even when the disclosure is negative.

Sample size	No statistical method was used to determine the sample size. Sample sizes were selected based on the experiment type and the standard practices in the field of genetics and stem cell biology (Miquerol et al. Circ. Res 107:153-161 (2010); Chabbab et al; Cell report 14:1-10 (2016). This approach allowed us to determine statistical differences within isogenic animal cohorts. For each experiment, we have used at least n=3 animals, and technical replicates with at least n=3 were used to calculate the statistical value of each analysis. The number of animals/ genotype used for each experiment is specified in the figure legends: globally, 78 Cx40CreERT2/R26R-confetti mice, 73 SmaCreERT2/R26R-confetti, 16 MespCre/R26RConfetti, 49 Cx40CreERT2/R26-YFP mice, 6 SmaCreERT2/R26-YFP, 10 Nkx2-5flox/R26-YFP/Cx40CreERT2 mice were used.
Data exclusions	No animal was excluded from this study.
Replication	All experiments have been repeated at least three times. The exact numbers are clearly indicated in the figures legends or table. All experimental findings were reliably reproduced.
Randomization	Randomization was not used for this study, which involved complex mouse genetics with mutiple transgenes.
Blinding	Data collection was not blinded as the phenotype was visible from the samples (opened ventricles). Clone analyses (size and type) were carried out blinded of sample genotyping.

Reporting for specific materials, systems and methods

We require information from authors about some types of materials, experimental systems and methods used in many studies. Here, indicate whether each material, system or method listed is relevant to your study. If you are not sure if a list item applies to your research, read the appropriate section before selecting a response.

Materials & experimental systems

n/a	Involved in the study
☐	☒ Antibodies
☒	☐ Eukaryotic cell lines
☒	☐ Palaeontology and archaeology
☐	☒ Animals and other organisms
☒	☐ Human research participants
☒	☐ Clinical data
☒	☐ Dual use research of concern

Methods

n/a	Involved in the study
☒	☐ ChIP-seq
☒	☐ Flow cytometry
☒	☐ MRI-based neuroimaging

Antibodies

Antibodies used	Antibody against: sheep GFP(AbdSerotec, Biorad)1:500; chick GFP 1020 (Aves) 1/500; Contactin-2 (AF1714, R&D Systems) 1:100 (sections) or 1/200 (wholemount); WGA-555 (Wheat-germ agglutinin, Clinisciences) 1:500. Donkey anti-goat-488 (A11055, Life technologies) 1:500 ; Donkey anti-chick-488 (703 545 155, Interchim) 1:500; Donkey anti-goat-647 (A21447, Life Technologies)1:250.
Validation	All commercial antibodies were validated by the manufacturer for the species and application used in the study (Immunohistochemistry).<br>The Sheep anti GFP antibody is recommended for immunofluorescence by the Manufacturer.<br>The Chick-anti GFP antibody was analyzed by Immunohistochemistry (1:500 dilution) using transgenic mice expressing the GFP gene product.<br>Contactin-2/TAG1was detected in immersion fixed paraffin-embedded sections of human brain (cortex) using Goat Anti-Human/ Mouse/Rat Contactin-2/TAG1 Antigen Affinity-purified Polyclonal Antibody (Catalog # AF1714) at 15 µg/mL overnight at 4 °C.

Animals and other organisms

Policy information about studies involving animals; ARRIVE guidelines recommended for reporting animal research

Laboratory animals	Adult mice, at least 8 weeks-old, were used for breeding and 3 weeks-old (P21) mice (males and females) for analyses.<br>Mouse line used were previously described:<br>-Cx40-CreERT2, Beyer, S., Kelly, R.G. & Miquerol, L. Inducible Cx40-Cre expression in the cardiac conduction system and arterial endothelial cells. Genesis 49, 83-91 (2011).<br>-Sma-CreERT2, Wendling, O., Bornert, J.M., Chambon, P. & Metzger, D. Efficient temporally-controlled targeted mutagenesis in smooth muscle cells of the adult mouse. Genesis 47, 14-18 (2009).<br>- Mesp1-cre, Saga, Y. et al. MesP1 is expressed in the heart precursor cells and required for the formation of a single heart tube. Development 126, 3437-3447 (1999).<br>- R26R-YFP, Srinivas, S. et al. Cre reporter strains produced by targeted insertion of EYFP and ECFP into the ROSA26 locus. BMC Dev Biol 1,4 (2001).<br>- R26-Confetti, Snippert, HJ et al. Intestinal crypt homeostasis results from neutral competition between symmetrically dividing Lgr5 stem cells. Cell 143, 134-144 (2010).<br>- Nkx2-5-LacZ, Tanaka, M et al. The cardiac homeobox gene Csx/Nkx2.5 lies genetically upstream of multiple genes for heart development. Development 126, 1269-1280 (1999).<br>- Nkx2-5-Floxed, Furtado, MB et al. A novel conditional mouse model for Nkx2-5 reveals transcriptional regulation of cardiac ion channels. Differentiation 91, 29-41 (2016).
Wild animals	This study did not involve wild animals.
Field-collected samples	This study did not involve field-collected samples.
Ethics oversight	All studies and procedures involving animals were in strict accordance with the recommendations of the European Community Directive (2010/63/UE) for the protection of vertebrate animals used for experimental and other scientific purposes. The project was specifically approved by the ethics committee of the IBDM SBEA and by the French Ministry of Research (APAFIS #01055.02).

Note that full information on the approval of the study protocol must also be provided in the manuscript.
